# Supplementary material for: Comparison of Fruit Texture and Storage Quality of Four Apple Varieties
Source: Foods. 2024 May 17;13(10):1563. doi: 10.3390/foods13101563 (PMC11121378; doi:10.3390/foods13101563)
Supplement: Supplementary file 1 [file foods-13-01563-s001.zip › foods-2993183-supplementary.pdf]

Table S1. The primers used for qRT-PCR

Primer name	Forward primer	Reverse primer
MdPL1	GTGCGGATCTTGCTACGAGATTAGG	GCTGTTGGATTGCCAGTTTT
MdPL22	GGTGCGGATCTTGCTACGAG	GCTGTTGGATTGCCAGTTTT
MdPG	CGGCTAGTGGCAGTTTGGTA	CGTGGAAGATTTGCTTGAGGG
MdXTH	GGCCAATGTGGGTTTATGGT	CCTGTACTGCTGTCGGGTCA
MdACO1	TTCCTGTTCCTCCTGATTCTT	CATGGTTCACAGCTCAAAG
MdACS1	AAATCCGAGGGAACAAAGTG	ATCAAATCCTGGGTAGTATGG
Md β-gal1	TCCTTGGCAGTCATTCATCG	TTCCCGTATTCAAACCCTTC
Md β-gal2	ACTCGGAAGTAATCAAGAGGC	TCTCCAATGACCCATACACG
MdEXP	TACCAGCAAGGGAAGAAAGA	TCAGAGGAACAACCCAAGTC
